#  

**DOI:** 10.6061/clinics/2019/e743err

**Published:** 2019-05-13

## CLINICS 2019;74:e743

In the article **The effectiveness of current informative material in improving awareness and opinion of undergraduate students towards organ donation: a comparative, randomized survey study**, on page 1, **the author’s name:**

“Ilka Santana de Fátima Ferreira Boin”

**should read:**

“Ilka de Fátima Santana Ferreira Boin”

**where it reads**

“Riccetto E, Boin IS. The effectiveness of current informative material in improving awareness and opinion of undergraduate students towards organ donation: a comparative, randomized survey study. Clinics. 2019;74:e743”

**it should read:**

“Riccetto E, Boin IF. The effectiveness of current informative material in improving awareness and opinion of undergraduate students towards organ donation: a comparative, randomized survey study. Clinics. 2019;74:e743”

**and on page 6, where it reads:**

## AUTHOR CONTRIBUTIONS

“Riccetto E was responsible for the study design, data collection, analysis/interpretation, statistics, manuscript drafting. Boin IS was responsible for the study design, data analysis/interpretation, manuscript drafting and critical revision.”

“Riccetto E was responsible for the study design, data collection, analysis/interpretation, statistics, manuscript drafting. Boin IF was responsible for the study design, data analysis/interpretation, manuscript drafting and critical revision.”

